# Improved Fibroblast Functionalities by Microporous Pattern Fabricated by Microelectromechanical Systems

**DOI:** 10.3390/ijms150712998

**Published:** 2014-07-22

**Authors:** Hongbo Wei, Lingzhou Zhao, Bangdao Chen, Shizhu Bai, Yimin Zhao

**Affiliations:** 1 State Key Laboratory of Military Stomatology, Department of Oral Implant, School of Stomatology, the Fourth Military Medical University, No. 145 West Changle Road, Xi’an 710032, China; E-Mail: weihongbo101@gmail.com; 2 State Key Laboratory of Military Stomatology, Department of Periodontology, School of Stomatology, the Fourth Military Medical University, No. 145 West Changle Road, Xi’an 710032, China; 3 State Key Laboratory for Manufacturing Systems Engineering, Xi’an Jiaotong University, Xi’an 710048, China; E-Mail: bangdao2008@sina.com; 4 State Key Laboratory of Military Stomatology, Department of Prosthodontics, School of Stomatology, the Fourth Military Medical University, No. 145 West Changle Road, Xi’an 710032, China; E-Mail: shizhu1976@gmail.com

**Keywords:** fibroblasts, microelectromechanical systems, percutaneous implant, microporous structure, surface modification

## Abstract

Fibroblasts, which play an important role in biological seal formation and maintenance, determine the long-term success of percutaneous implants. In this study, well-defined microporous structures with micropore diameters of 10–60 µm were fabricated by microelectromechanical systems and their influence on the fibroblast functionalities was observed. The results show that the microporous structures with micropore diameters of 10–60 µm did not influence the initial adherent fibroblast number; however, those with diameters of 40 and 50 µm improved the spread, actin stress fiber organization, proliferation and fibronectin secretion of the fibroblasts. The microporous structures with micropore diameters of 40–50 µm may be promising for application in the percutaneous part of an implant.

## 1. Introduction

The long-term success of the percutaneous implants relies on not only osseointegration but also a stable soft tissue biological seal [1,2,3]. The biological seal around the percutaneous part of an implant acts as a barrier to prevent bacterial invasion to the sub-epithelial connective tissues and the deeper area around the implant [1,4,5]. In fact, infection and loss of biological seal are the main reasons for percutaneous implant failure [1,3,6,7,8,9,10,11]. To enable faster soft tissue biological seal establishment and good maintenance, rational surface design for the percutaneous part of implants is deemed important.

Soft tissue mainly consists of fibroblasts that produce extracellular matrix (ECM) and various essential components of the connective tissues, such as glycosaminoglycan and collagen [12,13]. Hence, fibroblasts are believed to play an important role in biological seal formation and maintenance. The *in vitro* response of fibroblasts to an implant surface structure can be used as a suitable model to assess the implant’s ability to generate an ideal biological soft tissue.

In general, a smooth surface is considered suitable for formation of a stable biologic seal [5,14]. Upon healing, wound closure is likely to be generated by the contraction of fibrous connective tissues during the healing process. However, such smooth surfaces have been shown to lead to the creation of a detrimental capsule [5,11]. It was recently indicated by several reports that a suitable micro-roughened surface may be more effective for establishing a widely and tightly attached connective tissue seal [15,16,17]. However, the drawback of these techniques is that they offer only a limited control of the surface characteristics.

With the rapid development in the micro- and nanotechnology, it is now feasible to produce structures with well-defined shapes with up to nanometer resolution [18,19]. Microelectromechanical systems (MEMS), which can be basically described as the development of structures in the micro- and even nano-dimension using a micromachining process, were introduced in the late 1980s and have been adapted for biological and medical applications [20,21,22,23]. Over the last decades, a variety of micro-engineered architectures have been developed by MEMS, leading to significant advances in different fields of medicine and biology. Accordingly, we were particularly interested in fabricating precisely designed feature sizes and shapes on the implant surfaces by MEMS to determine whether these can improve the soft tissue seal for the percutaneous implant.

This study aims to evaluate the effect of precisely designed microporous structures manufactured by MEMS on fibroblast functionalities for possible application in the percutaneous part of an implant.

## 2. Results

### 2.1. Surface Characterization

**Figure 1:**
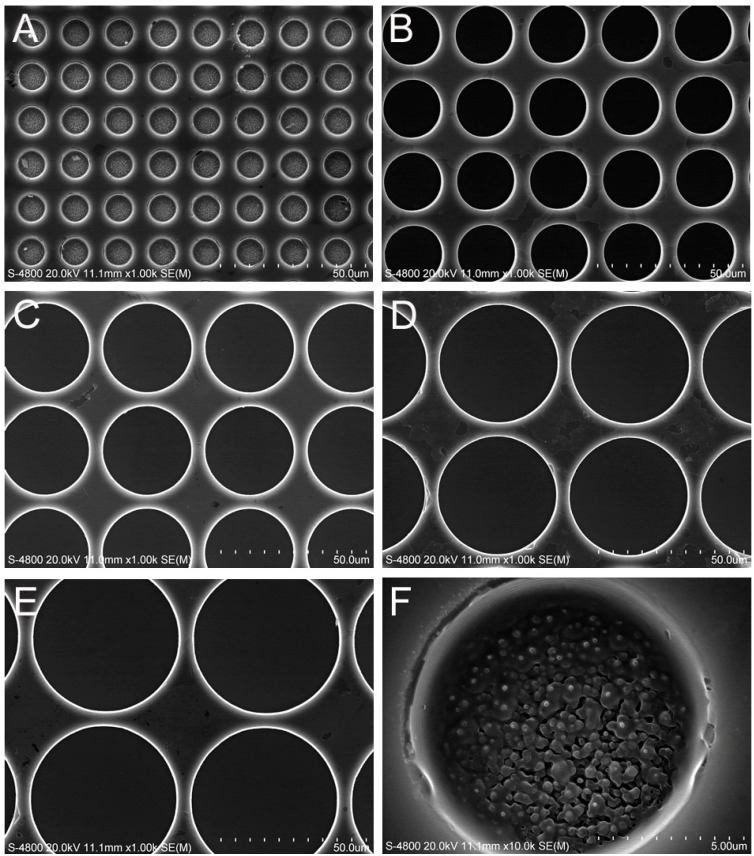
SEM images of the microporous structures with different micropore diameters: (**A**) 10 µm; (**B**) 20 µm; (**C**) 30 µm; (**D**) 40 µm; (**E**) 50 µm and (**F**) 60 µm.

The SEM micrographs shown in Figure 1 illustrate six microporous structures with highly ordered and vertically aligned micropores of different diameters, namely 10 µm (Figure 1A), 20 µm (Figure 1B), 30 µm (Figure 1C), 40 µm (Figure 1D), 50 µm (Figure 1E), and 60 µm (Figure 1F) which were fabricated by MEMS. The micropores on all of the substrates were approximately 10 µm in depth. The titanium film was deposited uniformly on the substrates.

### 2.2. Fibroblast Adhesion

**Figure 2:**
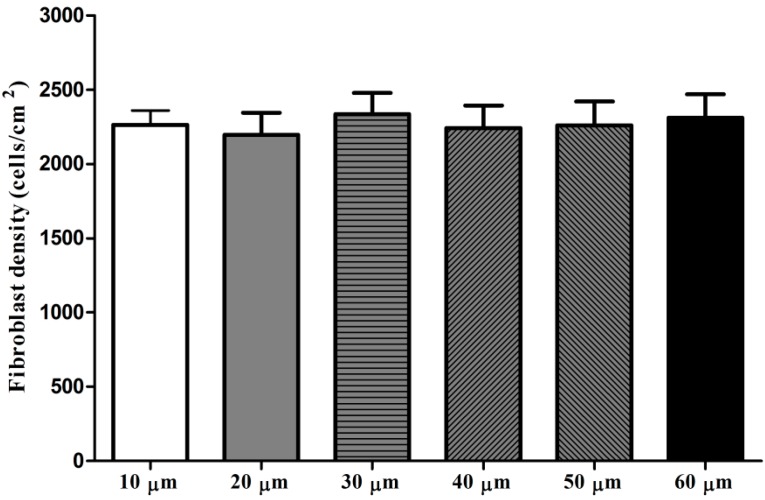
Fibroblast adhesion on microporous structures with different micropore diameters after 4 h of culture.

The adhesion assay results shown in Figure 2 indicate that there was no statistically significance in the adherent fibroblast numbers among the six microporous structures after 4 h of culture.

### 2.3. Fibroblast Proliferation

**Figure 3:**
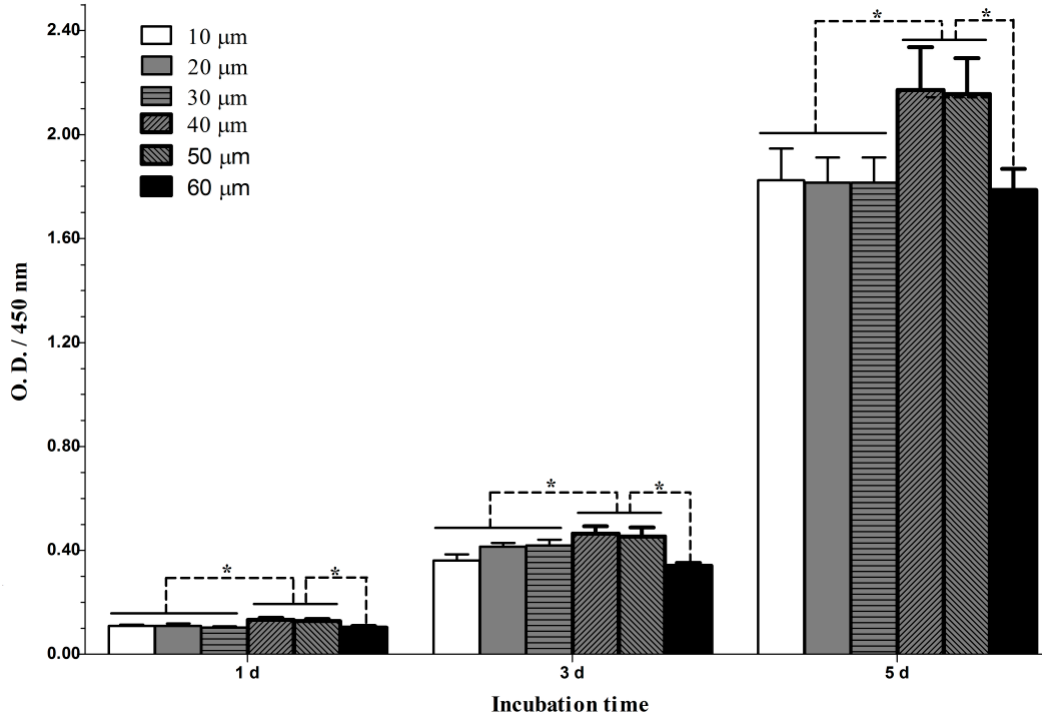
Cell proliferation on microporous structures with different micropore diameters. *n* = 6, * *p* < 0.05.

The proliferation of fibroblasts grown on the microporous structures is shown in Figure 3. The results show that the cells proliferated well over an incubation period from 1–5 days. In particular, the cells appeared to grow faster during the period of 3–5 days than the period of 1–3 days. At each time slot, the microporous structures with micropore diameters of 40 and 50 µm induced significantly better cell proliferation than the other structures. However, no significant difference was found in cell proliferation between the structures with micropore diameters of 40 and 50 µm and between those with diameters of 10, 20, 30 and 60 µm.

### 2.4. Fibroblast Morphology and Cytoskeletal Actin Organization

**Figure 4:**
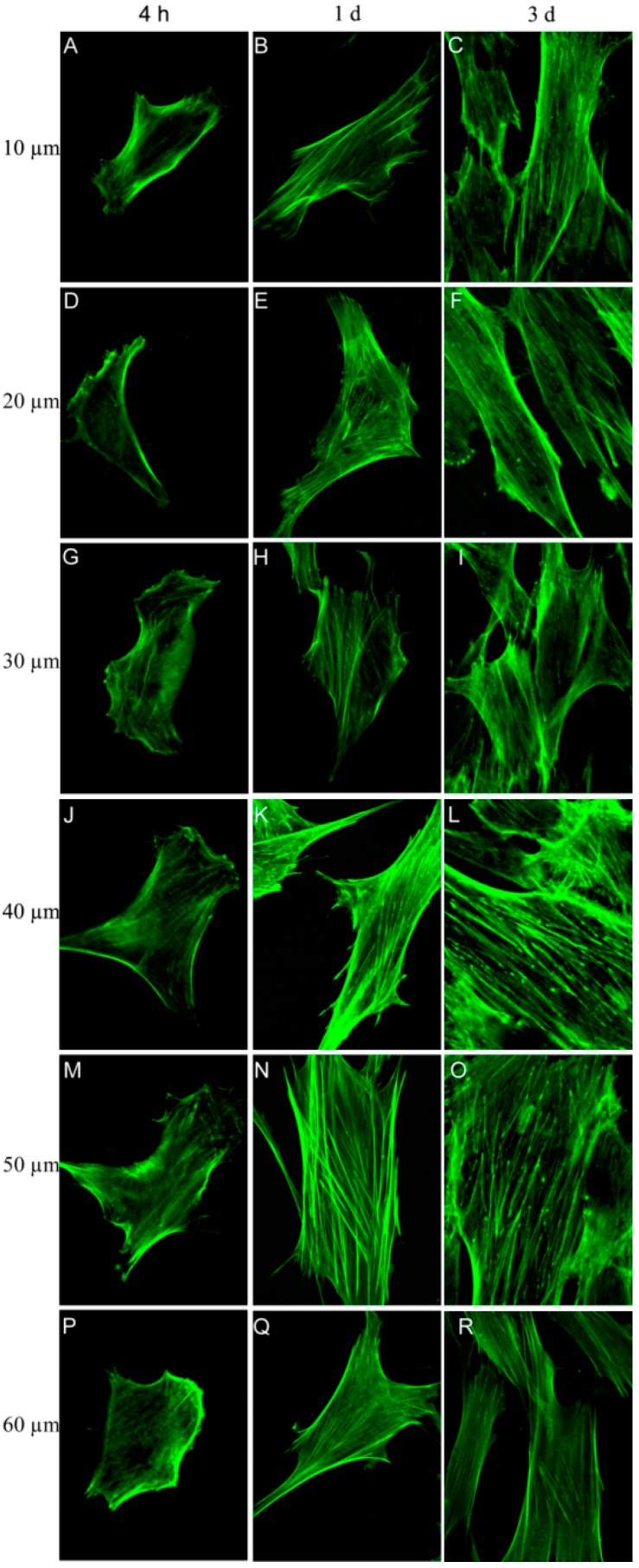
Fluorescence staining of the actin cytoskeleton of the fibroblasts cultured on microporous structures with different micropore diameters: (**A**–**C**) 10 µm; (**D**–**F**) 20 µm; (**G**–**I**) 30 µm; (**J**–**L**) 40 µm; (**M**–**O**) 50 µm; and (**P**–**R**) 60 µm.

The actin staining results show that the fibroblasts attached well to and spread well on the microporous structures (Figure 4). Notably, the cells on the microporous structures spread gradually during the incubation period from 4 h to 3 days. After 4 h, apparent stress fibers were observed around the periphery of the cells on the microporous structures with micropore diameters of 40 and 50 µm, but the cells on the other four structures showed nearly no stress fibers. At days 1 and 3, the cells on the microporous structures with micropore diameters of 40 and 50 µm displayed thick and contractile stress fibers, whereas those on the other four structures had noticeably fewer stress fibers. Up to day 3, the cells on the microporous structures with micropore diameters of 40 and 50 µm exhibited a well-spread cell body and nearly reached confluence, whereas the cells on the other structures were relatively less spread and far from confluence.

### 2.5. Fibronectin Secretion

**Figure 5:**
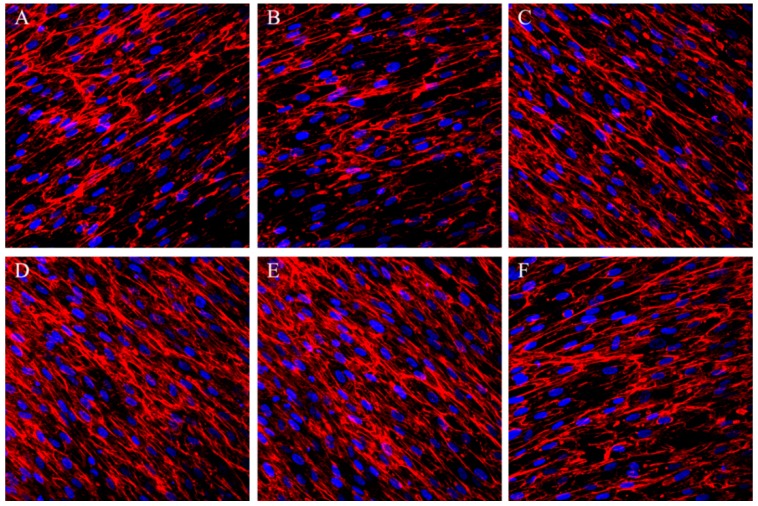
Immunofluorescence staining of fibronectin (red) and nuclei counterstaining, with DAPI (blue) of the fibroblasts cultured on the microporous structures with different micropore diameters: (**A**) 10 µm; (**B**) 20 µm; (**C**) 30 µm; (**D**) 40 µm; (**E**) 50 µm and (**F**) 60 µm.

As shown in Figure 5, on day 7, abundant fibronectin on all of the structures secreted by the fibroblasts with fibrillar distribution, but the fibronectin amounts were much higher on the microporous structures with micropore diameters of 40 and 50 µm as indicated by the denser fibronectin immunofluorescence signals.

## 3. Discussion

Infection and epithelial downgrowth are the major problems associated with percutaneous implants and both are mainly due to improper closure of the implant–soft tissue interface [24]. A rapidly established biological seal with long-term stability is then required for the percutaneous implants to perform well. There are various aspects affecting the biological seal, including the surgical techniques, the implant design and the implant surface morphology [24]. Regarding the implant surface morphology, the percutaneous surface of the currently used implants is often smooth to prevent the adhesion of bacteria [5,14]. However, the smooth surface has been shown to lead to a detrimental capsule and an unsatisfactory biological seal [5,11]. Many studies have aimed to prevent bacterial infection by fabricating antibacterial coatings [25,26], whereas few studies have concentrated on the biological seal of the percutaneous site. It has been indicated that a suitably micro-structured implant surface is promising for achieving more rigid biological seal [15,16,17]. We observed that the microporous structures with micropore diameters of 10–60 µm influenced the fibroblast functionalities and that those with 40 and 50 µm improved the viability, spread, actin stress fiber organization and fibronectin secretion of the fibroblasts. The microporous structures with micropore diameters of 40–50 µm may be promising for application in the percutaneous part of the implant.

The initial cell adhesion is considered to be the key step for the subsequent cell–biomaterial interaction as well as the final tissue integration. We evaluated the initial fibroblast adhesion on the microporous structures and found that the adherent cell numbers on the microporous structures with micropore diameters of 10–60 µm after 4 h of culture showed no obvious difference. Although he microporous structures showed no effect on the initial adherent cell number, the actin staining results displayed that the microporous structures with micropore diameters of 40 and 50 µm induced more rapid cell actin stress fiber organization as early as 4 h of culture, indicating that micropores with diameters of 40 and 50 µm may benefit the fibroblast functionalities.

After establishing stable attachment to the substrate, the cells will undergo the spread, proliferation and ECM secretion processes to form a more stable cell–substrate interaction. The microporous structures with micropore diameters of 40 and 50 µm induced significantly better cell proliferation, and culture times of up to 1 and 3 days resulted in denser and thicker parallel-oriented stress fibers on these microporous structures. We then inspected whether they also improve cell ECM secretion. ECM components may play an important role in cell–biomaterial and cell–cell interaction. Fibronectin is an important ECM that plays critical roles in cell survival, proliferation, attachment and differentiation [27]. We found that the cells secreted abundant fibronectin after 7 days of culture. The secreted fibronectin formed a denser extracellular mesh on the microporous structures with micropore diameters of 40–50 µm, indicating the formation of more stable cell–biomaterial and cell–cell interactions on these materials. The data from the cell proliferation, actin and fibronectin staining analyses are in good accordance, jointly demonstrating that the microporous structures with micropore diameters of 40–50 µm well promote various fibroblast functionalities including attachment, spread, growth and ECM synthesis and secretion.

The MEMS that can produce surfaces with well-defined feature sizes and shapes up to nanometer resolution provide a better method for inspecting the interactions between cells and substrates. Three-dimensional scaffolds have been previously prepared by MEMS to study the interaction between cell and material [28,29,30], but for titanium implants, we are more concerned with the surface topography, which influences the behavior of the cells growing on it. The microporous pattern that we need can be easily produced on a silicon wafer, and a Ti film with a thickness of 100 nm was then deposited on the silicon wafer to simulate the surface of the titanium implant. In this study, the microporous structures with micropore diameters of 40 and 50 µm resulted in improved fibroblast functionalities and are thus promising for application in the percutaneous part implants. Nonetheless, more studies such as investigations of their influence on the functionalities of the epithelium, which is another important component of the biological seal, and *in vivo* animal experiments are necessitated to draw a final conclusion.

## 4. Experimental

### 4.1. Precisely Designed Microporous Structure Manufactured by MEMS

**Figure 6:**
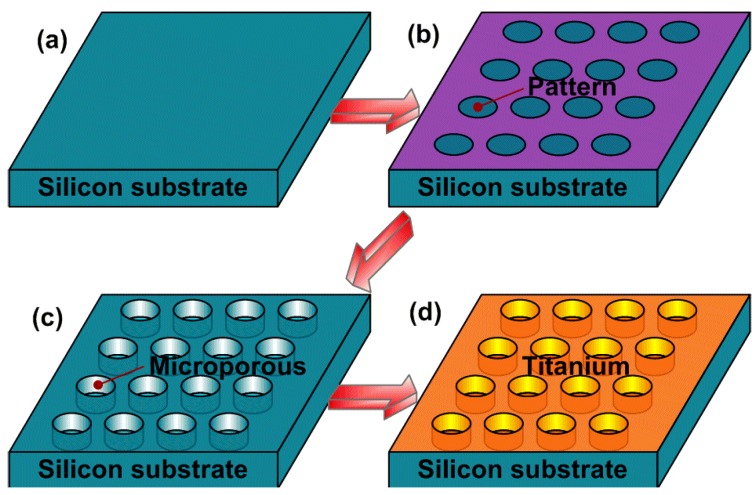
The manufacture process of the microporous surface by MEMS: (**a**) silicon substrate; (**b**) photolithography process; (**c**) deep reactive ion etching; (**d**) magnetron sputtering deposition.

A modified Bosch process was used to manufacture the precisely designed microporous structure [31], and this process is schematically shown in Figure 6. All of the patterns for the microporous structures with different diameters were defined by a photolithography process on one silicon substrate (Figure 6b). The silicon wafer was then treated by deep reactive ion etching (DRIE) for 10 min to form the microporous structure (Figure 6c). Finally, a titanium (Ti) film with a thickness of 100 nm was deposited on the silicon wafer by magnetron sputtering deposition (Figure 6d).The sheets were then cut into squares of 10 × 10 mm^2^, immediately rinsed with abundant deionized water, air-dried and sterilized under ultraviolet light for 3 h per side prior to the cell culture experiments. The structural characterization of these surfaces was performed using a field emission scanning electron microscope (SEM, S4800, Hitachi, Japan).

### 4.2. Fibroblast Culture

Primary human skin fibroblasts were isolated and cultured as previously described [32]. The fibroblasts were cultured in Dulbecco’s Modified Eagle Medium (DMEM, HyClone, UT, USA) with 10% fetal bovine serum (FBS, HyClone, UT, USA) and 1% penicillin/streptomycin (Sigma, St. Louis, MO, USA) at 37 °C in a humidified condition with 5% CO_2_ and 95% air. The cells were used at passages 3–8. For different assays, the cells were seeded onto the experimental substrates and placed in the 48-well polystyrene cell culture plate, at a density of 3500 cells/cm^2^ for the cell adhesion assay and at a density of 1000 cells/cm^2^ for the other assays. The cell culture media were replaced every two days.

### 4.3. Cell Adhesion Assay

After 4 h of culture, the substrates were rinsed in phosphate buffered saline (PBS) to remove any non-adherent cells. The adherent cells on the substrates were fixed with formaldehyde for 15 min, stained with 4,6-diamidino-2-phenylindole (DAPI, Sigma, St. Louis, MO, USA) for 5 min, and consequently counted under a fluorescence microscope (DMI6000B, Leica, Wetzlar, Germany). The cells in five random fields were counted per substrate.

### 4.4. Fibroblast Proliferation Assay

The cell proliferation was assayed with a cell counting kit-8 (CCK-8, Dojindo Molecular Technologies, Kumamoto, Japan) according to the manufacturer’s instruction. Briefly, after 1, 3 and 5 days of culture, the culture media were removed and 200 mL of fresh culture medium and 20 µL of CCK-8 reagent were added into each well for 2 h of incubation. The same protocol was conducted on the culture plate with no seeded cells as the background control. Aliquots of 150 mL from the incubated medium were pipetted into a 96-well plate and the absorbance at 450 nm was measured. The experiment was performed with a sample size of *n* = 6.

### 4.5. Fluorescent Staining of Cytoskeletal Actin

Actin fluorescent staining was used to display both the organization of the actin cytoskeleton and the cellular shape. After 4 h and, 1 and 3 days of culture, the cells on the substrates were washed with PBS, fixed in a 4% paraformaldehyde solution for 15 min, washed again with PBS and treated with 0.1% Triton X-100 for 5 min at room temperature. After incubation with 1% bovine serum albumin (BSA)/PBS at 37 °C for 5 min and then washing with PBS, the samples were incubated with phalloidin-FITC for 1 h at 37 °C and then visualized under a laser scanning confocal microscope (FV1000, Olympus, Tokyo, Japan).

### 4.6. Fibronectin Secretion Assay

Immunofluorescent staining of fibronectin which is hypothesized to play an important role in mediating the cell adhesion to biomaterials was conducted. After 7 days of culture, the cells were fixed with 4% paraformaldehyde, washed with PBS and treated with 0.1% Triton X-100 at room temperature for 5 min. Afterwards, the samples were incubated in 1% BSA/PBS at 37 °C for 5 min, washed with PBS and incubated with the anti-fibronectin mouse antibody (B&D, Macon, GA, USA) for 2 h at room temperature. Then, a Cy3-conjugated goat anti-mouse secondary antibody (B&D, USA) was added, and the culture was incubated 1 h. Finally, laser scanning confocal microscopy was used to visualize the stained fibronectin.

### 4.7. Statistical Analyses

All of the experiments were repeated at least three separate times. Analysis of variance (ANOVA) followed by Student-Newman-Keuls *post hoc* test were used to determine the statistical significance. *p* < 0.05 was considered to be significant.

## 5. Conclusions

Well-defined microporous structures with micropore diameters of 10–60 µm can be fabricated by microelectromechanical systems, which provide a good platform to study the interaction of fibroblasts with biomaterial topography. The microporous structures with different micropore diameters of 10–60 µm do not induce an obvious influence in the initial adherent fibroblast number; however, those with diameters of 40 and 50 µm significantly improve the fibroblast functionalities including their spread, actin stress fiber organization and proliferation, and ECM secretion. The microporous structures with micropore diameters of 40–50 µm show great potential for application in the percutaneous part of implants, but further study concerning their influence on the functionalities of the epithelium, and *in vivo* animal experiments are still necessary.
